# B[μ-H(CO_3_)_2_]: An Acentric Boron Hydrogencarbonate with [μ-H(CO_3_)_2_]^3–^ Complex Anions

**DOI:** 10.1021/acs.inorgchem.5c03153

**Published:** 2025-09-16

**Authors:** Dominik Spahr, Tim H. Reuter, Elena Bykova, Lkhamsuren Bayarjargal, Lukas Brüning, Valentin Kovalev, Maxim Bykov, Lena Wedek, Victor Milman, Jonathan Wright, Björn Winkler

**Affiliations:** † Institute of Geosciences, 9173 Goethe University Frankfurt, Altenhöferallee 1, 60438 Frankfurt, Germany; ‡ Institute of Inorganic and Analytical Chemistry, 9173 Goethe University Frankfurt, Max-von-Laue-Straße 7, 60438 Frankfurt, Germany; ¶ Dassault Systèmes BIOVIA, 334 Cambridge Science Park, Cambridge CB4 0WN, United Kingdom; § 55553 European Synchrotron Radiation Facility ESRF, 71 avenue des Martyrs, CS40220, 38043 Grenoble Cedex 9, France

## Abstract

The hydrous borocarbonate B­[μ-H­(CO_3_)_2_] was synthesized in a laser-heated diamond anvil cell at moderate pressures (∼20 GPa) and temperatures (∼1500(200) K) by a reaction between B_2_O_3_, CO_2_ and H_2_O. The crystal structure was obtained from synchrotron single-crystal X-ray diffraction and confirmed by a combination of density functional theory (DFT) calculations and Raman spectroscopy. Second harmonic generation (SHG) measurements corroborated the acentric space group, while DFT calculations provided the complete SHG tensor. In the [μ-H­(CO_3_)_2_]^3–^ building block two [CO_3_]^2–^ groups are connected by sharing a hydrogen atom, which is bound in a nearly linear and symmetric O–H–O arrangement. B­[μ-H­(CO_3_)_2_] is a hydrous borocarbonate without further cations, combining interesting chemical and physical properties such as symmetric hydrogen bonds and a significant SHG intensity.

## Graphical abstract

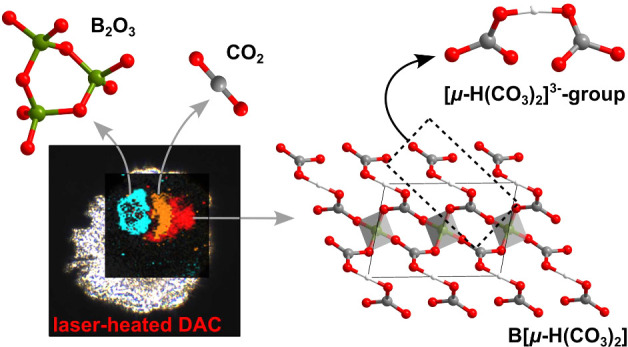

Borates, containing boron oxoanions, are a well-established, structurally very diverse class of optical materials which are employed in numerous applications.^1–5^ While chemically complex boron-containing compounds hosting multiple anionic units such as borosulfates, borophosphates or borosilicates have been studied extensively, chemically simple borocarbonates, i.e., carbonates with only boron as a cation and no anions other than carbonate groups, have not been described before now. Such borocarbonates would be interesting, as the trivalent boron cation B^3+^ is one of the smallest cations which can be present within a crystal structure (*r*(B^3+^) = 0.11 Å in 4-fold coordination).^6^ Its ionic radius is half of that of beryllium (*r*(Be^2+^) = 0.27 Å) and a third of that of Al (*r*(Al^3+^) = 0.39 Å).^6^

Whether or not a chemically simple borocarbonate can be obtained is a nontrivial question, as it is well established that boron will readily form complex borate anions such as [BO_3_]^3–^, [BO_4_]^5–^, [B_2_O_5_]^4–^, [B_3_O_6_]^3–^ or [B_5_O_10_]^5–^.^1^ For more complex chemical compositions, carbon-containing borates such as potassium bis­(carbonato)­borate hydrate (K­[B­(CO_2_-μ-O-CO_2_)_2_]·2H_2_O), where a boron atom is in the center of a complex surrounded by two [C_2_O_5_]^2–^ groups have been described.^7^ Furthermore, borate carbonates hosting carbonate anions, as well as borate anions, are known (e.g., Ba_5_[CO_3_]_2_[BO_3_]_2_ or Ca_4_Mn_3_[[BO_3_]_3_[CO_3_]­O_3_]).^8,9^ Also, borate oxalates without fluorine anions (e.g., *A*[B­(C_2_O_4_)_2_] with *A* = K, Na, Li) have been synthesized.^10^ In the B-C-O-H system, compounds such as B­(OCH_3_)_3_ or B_3_H_7_(CO) have been obtained, but no crystal structure of a chemically simple borocarbonate without other cations has been described.^11,12^ In naturally occurring boron minerals, B^3+^ cations are 3- or 4-fold coordinated. For B_2_O_3_ a pressure-induced phase transition at 2–6 GPa, depending on temperature,^13^ leads to an increase in the coordination from three to four, in agreement with the “pressure coordination rule”.^14–17^ It is therefore unclear which coordination the B^3+^ cations would adopt in a hypothetical borocarbonate.

Here we investigated the system B_2_O_3_ + CO_2_ (+ H_2_O) in order to understand if a chemically simple borocarbonate can be synthesized, in analogy to Al_2_[CO_3_]_3_ and Be­[CO_3_], or if more complex hydrous carbonates (i.e., in analogy to Li­[HC_2_O_5_]) will form.^18–21^ In previous experiments, we found that sometimes small amounts of H_2_O from the CO_2_ gas co-condense into the gasket hole during the cryogenic loading.^21^ B_2_O_3_ was compressed to ∼20 GPa in the CO_2_ atmosphere in a laser-heated diamond-anvil cell (LH-DAC) (Figure 1 a). During pressure increase a phase transition from CO_2_-I to CO_2_-III (*Cmca*) occurs in a broad (∼5 GPa) pressure range around ∼12 GPa.^22–24^ The experimental Raman spectrum of CO_2_-III before the laser-heating at 20(2) GPa is accurately reproduced by the Raman spectrum obtained from our density functional theory (DFT) calculations (Figure 2 a). At pressures above ∼12 GPa heating of CO_2_-III causes the appearance of the high-temperature CO_2_ polymorphs CO_2_-II and CO_2_-IV depending on the temperature.^24–26^

**Figure 1:**
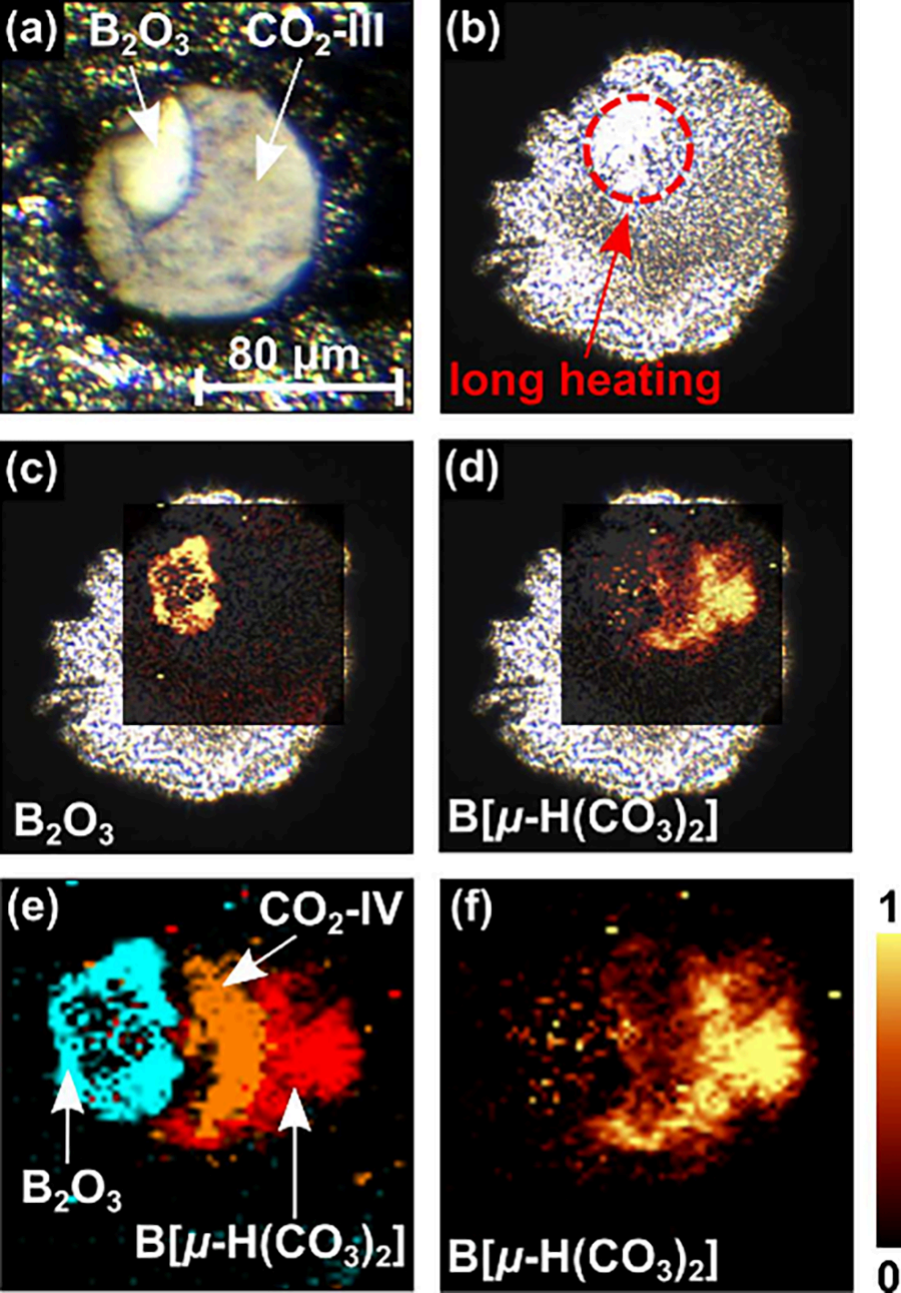
(a) B_2_O_3_ surrounded by CO_2_-III in the sample chamber of the DAC at ∼20 GPa before the laser-heating. (b) Sample chamber after laser-heating (*T*_max_ ≤ 1500(200) K). Raman maps overlaid on a picture of the sample chamber: (c) B_2_O_3_-*Ccm*2_1_ (∼390 cm^–1^) and (d) B­[μ-H­(CO_3_)_2_] (∼1225 cm^–1^). (e) Combined Raman maps of B_2_O_3_-*Ccm*2_1_, CO_2_-IV and B­[μ-H­(CO_3_)_2_]. (f) Raman map of B­[μ-H­(CO_3_)_2_] (∼1225 cm^–1^) at 20(2) GPa.

**Figure 2:**
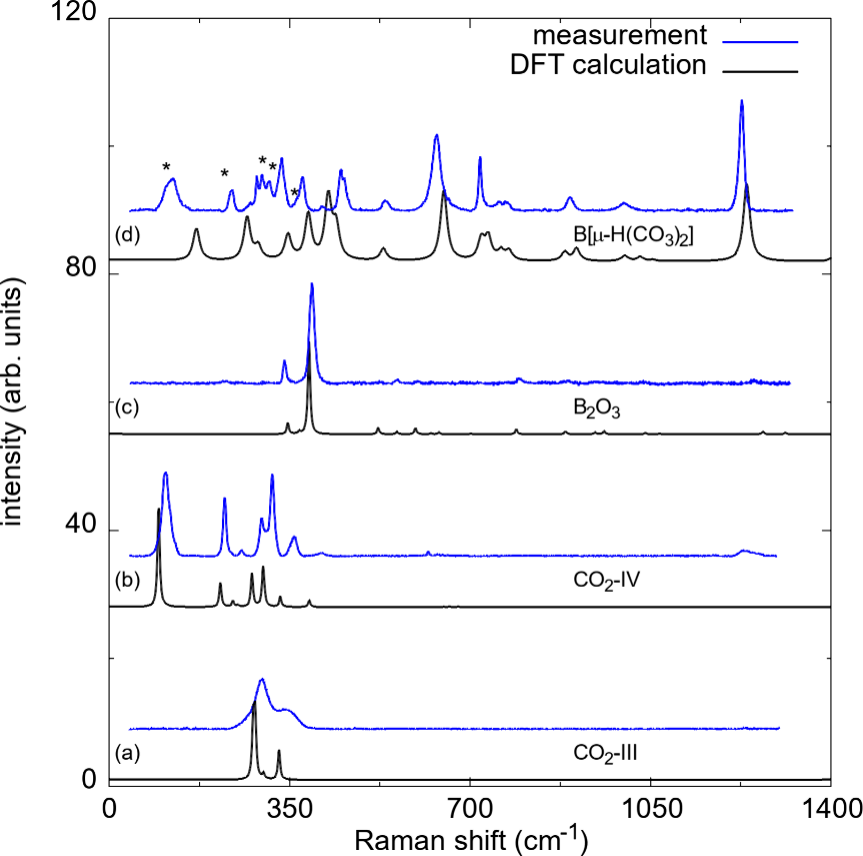
(a) Raman spectra for CO_2_-III at 20(2) GPa before the laser-heating. Raman spectra after laser-heating at 20(2) GPa (*T*_max_ ≤ 1500(200) K): (b) CO_2_-IV, (c) B_2_O_3_-*Ccm*2_1_ and (d) B­[μ-H­(CO_3_)_2_]. Experimental Raman spectra are shown in blue; DFT-based calculations are shown in black. The Raman shifts of the theoretical spectra were scaled by 1–2%. Peak positions of CO_2_-IV in the Raman spectrum of B­[μ-H­(CO_3_)_2_] are marked by an asterisk (*).

After the laser-heating (*T*_max_ ≤ 1500(200) K), we observed the characteristic Raman modes of CO_2_-IV. The experimental Raman spectrum of CO_2_-IV is in reasonable agreement with our DFT-based calculations (Figure 2 b). In addition, we observed the Raman modes of B_2_O_3_-II in the region where the B_2_O_3_ sample can be visually identified (Figure 1 c). The formation of B_2_O_3_-II was observed above 2–6 GPa, depending on temperature, in earlier studies, and this phase is predicted to be the stable polymorph up 46 GPa.^13,15,16^ The DFT calculations based on B_2_O_3_-*Ccm*2_1_ accurately reproduce the experimental Raman spectrum of B_2_O_3_-II (Figure 2 c).

Using spatially resolved Raman spectroscopy, we found an unknown phase in the sample chamber after heating (Figure 1 d–f). A strong Raman mode occurs at ∼1225 cm^–1^, which is typical for the C–O stretching mode in a [CO_3_]^2–^ group in carbonates hosting small cations such as Li_2_[CO_3_] or Be­[CO_3_].^18,27^ The B_2_O_3_ crystal was then laser-heated for ∼30 min (Figure 1 b, *T*_max_ ≤ 1500(200) K) to obtain a Raman spectrum with very little contamination by other phases (Figure 2 d). From the location of the new phase in the Raman maps next to the heated area (Figure 1 e), we infer that significantly lower temperatures are already sufficient for the formation of the new phase.

We determined the crystal structure of the unknown phase and found that it is the hydrous borocarbonate B­[μ-H­(CO_3_)_2_] (Figure 3 a). At 20(2) GPa B­[μ-H­(CO_3_)_2_] crystallizes in the monoclinic space group *C*2 with *Z* = 2 and *a* = 6.997(2) Å, *b* = 3.868(7) Å, *c* = 5.0197(8) Å and β = 101.14(3)° (*V* = 133.3(2) Å^3^). The experimental structural model is fully supported by the one obtained from our DFT-based full geometry optimizations (Table S1). It is generally accepted that DFT model calculations can reliably predict hydrogen positions.^28^ Hence we introduced a restraint for the O–H bond distance to the value derived from our DFT-based calculations in the refinement (∼1.2 Å). Without this restraint, the experimental O–H bond becomes slightly longer due to a small displacement of the H atom along the [010] direction. It should be stressed that the position of the hydrogen atom could be directly located in the difference Fourier map during the structure determination (Figure 4 b). Furthermore, we obtained a theoretical Raman spectrum from our DFT-based calculations, which reproduces the experimentally obtained Raman spectra reasonably well (Figure 2 d).

**Figure 3:**
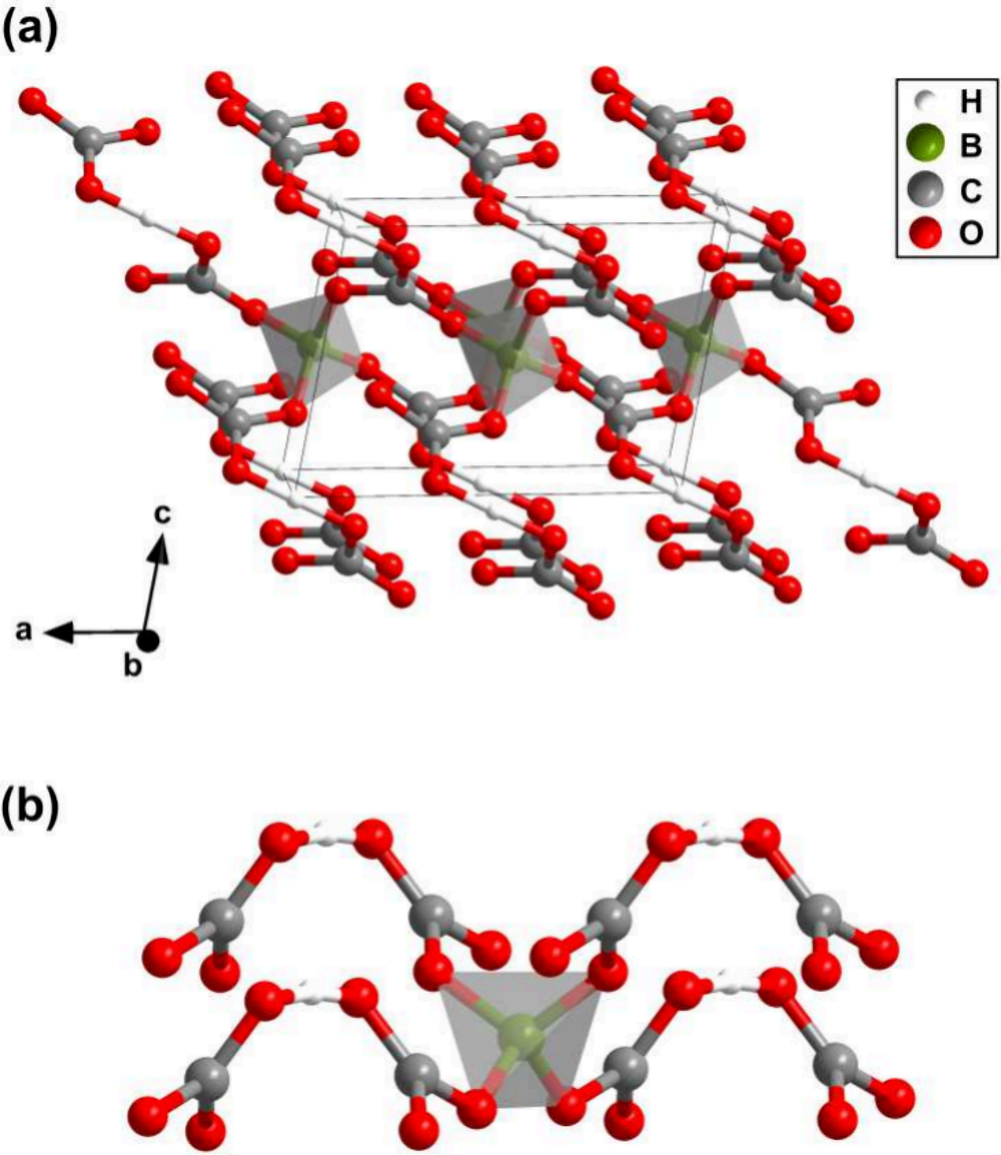
(a) Monoclinic structure (space group *C*2 with *Z* = 2) of B­[μ-H­(CO_3_)_2_]. (b) Coordination of a [BO_4_] tetrahedron by four [μ-H­(CO_3_)_2_]^3–^ complex anions. The structure was obtained from a single-crystal structure solution at 20(2) GPa.

**Figure 4:**
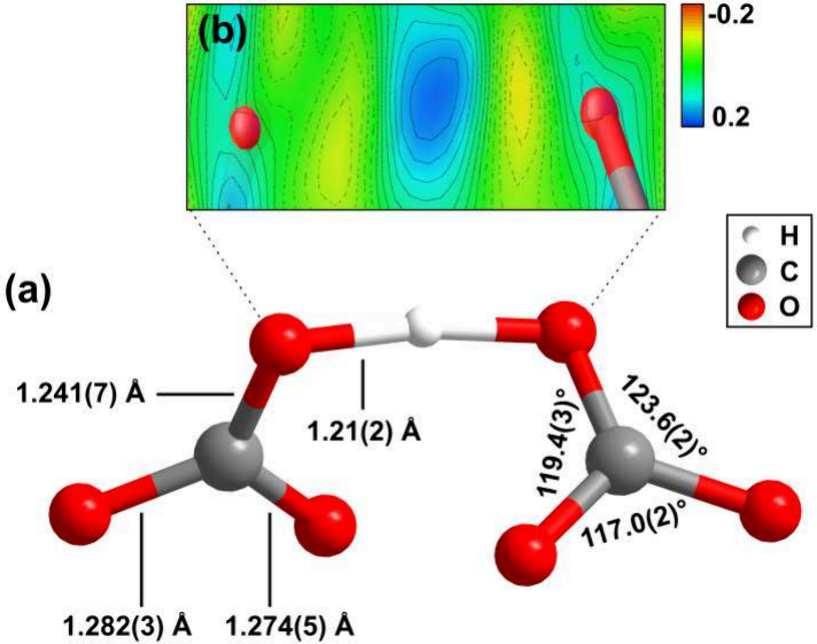
(a) Geometry of the [μ-H­(CO_3_)_2_]^3–^ complex anion in B­[μ-H­(CO_3_)_2_] at 20 GPa. (b) Difference Fourier map around the O–H–O bond at 20(2) GPa from single-crystal structure refinement without the hydrogen atom, showing the lack of a hydrogen atom located between the oxygen atoms.

The crystal structure of B­[μ-H­(CO_3_)_2_] is characterized by [BO_4_] tetrahedra with B–O bond lengths of 1.43(1) and 1.45(1) Å. The DFT calculations show that the bonds have a significantly covalent character with bond populations of 0.60 e^–^/Å^3^. The B–O bond length is in agreement with the distance in the [BO_4_] tetrahedra in B_2_O_3_-*Ccm*2_1_ (1.38–1.51 Å) and its distance in more complex compounds such as the hydrous borosulfate Sr­[B_3_O­(SO_4_)_4_(SO_4_H)] (1.46–1.52 Å) or the borophosphate U_2_[BO_4_]­[PO_4_] (1.46–1.49 Å).^15,29,30^ Each of the [BO_4_] tetrahedra in B­[μ-H­(CO_3_)_2_] is coordinated by four [μ-H­(CO_3_)_2_]^3–^ groups (Figure 3 b).

A second building block of the crystal structure is the [μ-H­(CO_3_)_2_]^3–^ group with a symmetric O–H–O arrangement. In these complex anions, two [CO_3_]^2–^ groups are connected by sharing one hydrogen atom. The [μ-H­(CO_3_)_2_]^3–^ groups can be considered to be an ordered and symmetric version of the [HC_2_O_6_]^3–^ group, described previously in the hydrous mineral trona (Na_3_[CO_3_]­[HCO_3_]·2H_2_O), in which hydrogen is distributed over two lattice sites.^31,32^ In contrast to the flat geometry of the disordered [HC_2_O_6_]^3–^ group in Na_3_[CO_3_]­[HCO_3_]·2H_2_O, here the [CO_3_]^2–^ groups are in the trans-configuration, bound by sharing a single hydrogen atom and twisted by an angle of ∼70° from the cis-configuration in B­[μ-H­(CO_3_)_2_] (Figure 4 a). This points toward a large flexibility of the [μ-H­(CO_3_)_2_]^3–^ hydrogen carbonate anion. Such twisting around a bridging hydrogen atom has also been observed between the [NO_3_]^−^ groups in the hydrogen dinitrate ion in Cs­[H­(NO_3_)_2_] by 75°.^33^

There is a slight deformation of the [CO_3_]^2–^ group due to the hydrogen bond. At 20(2) GPa C–O bond distances to the terminal oxygen atoms are 1.282(3) and 1.275(5) Å, while the distance to the oxygen atom which is connected to the hydrogen atom is shorter (1.241(7) Å). A Mulliken population analysis shows that along this latter bond the Mulliken population (0.95 e^–^/Å^3^) is larger than for the bonds to the terminal oxygen atoms (0.82 e^–^/Å^3^). The DFT calculations were carried out in space group *P*1 and yielded O–H bonds which are equally long (1.19 Å), with a Mulliken population of 0.95 e^–^/Å^3^. Such a bridging hydrogen atom with a symmetric character has, e.g., been observed in *Ln*[(μ_2_-H)­(H-mpdc)_2_]­(H-mpdc)_2_ (mpdc = 2,6-dimethylpyridine-3,5-dicarboxylate; *Ln* = Nd, Eu or Ce) at ambient conditions.^34^ Here, the O–H bonds range between 1.247 and 1.257 Å and the O–H–O angles are between 165 and 180°. The symmetrized hydrogen bond is also similar to the O–H–O geometry in δ-AlOOH at 18 GPa or in H_2_O ice-X at pressures ≥60 GPa.^35,36^

Our DFT-based calculations show that at pressures above ∼9 GPa the O–H–O geometry is symmetrical with two essentially identical O–H bond distances of 1.19 Å at 20 GPa (Figure S2). At lower pressures a phase transition occurs into a triclinic phase and the O–H–O geometry becomes asymmetric (Figure S2), with a clear distinction between the acceptor and the donor oxygen atoms (O···H–O). According to a long-established empirical relation an O–H···O distance of ∼2.45 Å should lead to an O–H stretching frequency of ∼1850 cm^–1^.^37^ Our calculations yield essentially pure O–H stretching vibrations at 1841 and 1903 cm^–1^ (Figure S5).

We used the DFT calculations to obtain the *p*, *V* relation for B­[μ-H­(CO_3_)_2_] and fitted the *p*, *V* data with an equation of state (EoS) (Figure S3). We obtained a bulk modulus of *K*_0_ = 24.4(6) GPa with *K*_p_ = 6.1(1). We also obtained the bulk modulus from stress–strain calculations, i.e., from the elastic stiffness tensor (Table S3). This approach yields *K*_0_ = 18.0(4) GPa, confirming that B­[μ-H­(CO_3_)_2_] is very compressible. Our DFT-based calculations reveal a relatively high second harmonic generation (SHG) coefficient for B­[μ-H­(CO_3_)_2_] (*d*_eff_ = 1.8 pm V^–1^) at 20 GPa (see SI). The calculated SHG coefficient is significantly higher than for quartz (*d*_eff_ = 0.2 pm V^–1^) at ambient conditions or for Be­[CO_3_] (*d*_eff_ = 1.3 pm V^–1^) at 20 GPa.^18^ Hence, we performed a second experiment in which the SHG signal was measured in the DAC at 20(2) GPa before and after the laser-heating. Before the laser-heating, we could not detect a SHG signal from the unreacted precursors using a laser defocused to approximately the size of the sample chamber (Figure S4). This is in contrast to the noticeable SHG signal (∼25 mV) from B­[μ-H­(CO_3_)_2_] after the laser-heating (Figure S4), confirming the acentric space group symmetry. 2D Raman mapping after laser-heating shows no peaks of unreacted B_2_O_3_, revealing a complete reaction.

In summary, we obtained the hydrous borocarbonate B­[μ-H­(CO_3_)_2_] which contains a fully ordered [μ-H­(CO_3_)_2_]^3–^ group. The [μ-H­(CO_3_)_2_]^3–^ group consists of two [CO_3_]^2–^ groups bridged by a hydrogen atom, which is part of a symmetric and nearly linear O–H–O arrangement at high pressures. On pressure decrease, DFT predicts a phase transition which leads to a conventional hydrogen bond. The acentric space group symmetry of the crystal structure was experimentally corroborated by SHG measurements and DFT-based calculations. B­[μ-H­(CO_3_)_2_] is the carbonate hosting the smallest cation described to date. The present study extends the family of *sp* ^2^-carbonates with trivalent metal cations, as we now have obtained Al_2_[CO_3_]_3_, Al_2_[C_2_O_5_]­[CO_3_]_2_, Fe_2_[CO_3_]_3_, Cr_2_[CO_3_]_3_ and B­[μ-H­(CO_3_)_2_].^20,38,39^ The twisting of the [CO_3_]^2–^ groups around the bridging hydrogen atom points toward a significant structural flexibility of the anion, and hence it is reasonable to assume that the [μ-H­(CO_3_)_2_]^3–^ group can be incorporated in a variety of structure types.

## Supplementary Material



## Notes

Experimental and computational details of the experiments and the results of the single-crystal structure solution, SHG measurements and DFT-based calculations (PDF)

## References

[1] Mutailipu M., Poeppelmeier K. R., Pan S. (2021). Borates: A Rich Source for Optical
Materials. Chem. Rev..

[2] Becker P. (1998). Borate Materials
in Nonlinear Optics. Adv. Mater..

[3] Chen C. T., Liu G. Z. (1986). Recent Advances
in Nonlinear Optical and Electro-optical
Materials. Annu. Rev. Mater. Sci..

[4] Mutailipu M., Zhang M., Yang Z. H., Pan S. L. (2019). Targeting the Next
Generation of Deep-Ultraviolet Nonlinear Optical Materials: Expanding
from Borates to Borate Fluorides to Fluorooxoborates. Acc. Chem. Res..

[5] Sasaki T., Mori Y., Yoshimura M., Yap Y. K., Kamimura T. (2000). Recent Development
of Nonlinear Optical Borate Crystals: Key Materials for Generation
of Visible and UV Light. Mater. Sci. Eng. R
Rep..

[6] Shannon R. D. (1976). Revised
effective ionic radii and systematic studies of interatomic distances
in halides and chalcogenides. Acta Crystallogr..

[7] Tombul M., Turkmenoglu E., Sahin O. (2021). Unprecedented Formation of Potassium
Borate Based Carbonate from Chloral Hydrate, Potassium Carbonate and
Boric Acid. X-Ray Struct. Anal. Online.

[8] Zhang X., Wu H., Yu H., Yang Z., Pan S. (2019). Ba_4_M­(CO_3_)_2_(BO_3_)_2_ (*M*=Ba, Sr): two borate-carbonates synthesized by
open high temperature
solution method. Sci. China Mater..

[9] Hoffmann C., Armbruster T., Kunz M. (1997). Structure refinement of (001) disordered
gaudefroyite Ca_4_Mn_3_
^3+^[(BO_3_)_3_(CO_3_)­O_3_]: Jahn-Teller-distortion in edge-sharing chains of
MnMn_3_
^3+^O_6_ octahedra. Sci. China. Mater..

[10] Zavalij P. J., Yang S., Whittingham M. S. (2003). Structures
of potassium, sodium and
lithium bis­(oxalato)­borate salts from powder diffraction data. Acta Crystallogr..

[11] Hartl M. A., Williams D. J., Acatrinei A. I., Stowe A., Daemen L. L. (2007). The Crystal
Structure of Trimethyl Borate by Neutron and X-ray Powder Diffraction. Z. Anorg. Allg. Chem..

[12] Glore J. D., Rathke J. W., Schaeffer R. (1973). Studies of Boranes. XXXVII. Some
Reactions of Triborane(7) and the Structure of Triborane(7)-Carbonyl. Inorg. Chem..

[13] Solozhenko V. L., Kurakevych O. O., Le Godec Y., Brazhkin V. V. (2015). Thermodynamically
Consistent *p*––*T* Phase
Diagram of Boron Oxide B_2_O_3_ by in Situ Probing
and Thermodynamic Analysis. J. Phys. Chem. C.

[14] Schreyer W., Werding G. (1997). High-pressure behaviour
of selected boron minerals
and the question of boron distribution between fluids and rocks. Lithos.

[15] Prewitt C. T., Shannon R. D. (1968). Crystal structure of a high-pressure
form of B_2_O_3_. Acta Crystallogr..

[16] Dong H., Oganov A. R., Brazhkin V. V., Wang W., Zhang J., Davari Esfahani M. M., Zhou X.-F., Wu F., Zhu Q. (2018). Boron oxides
under pressure: Prediction of the hardest oxides. Phys. Rev. B.

[17] Neuhaus A. (1964). Synthese Strukturverhalten
und Valenzzustände der anorganischen Materie im Bereich hoher
und höchster Drücke. Chimia.

[18] Spahr D., Bayarjargal L., Bykova E., Bykov M., Reuter T. H., Brüning L., Jurzick P. L., Wedek L., Milman V., Wehinger B., Winkler B. (2024). Synthesis and crystal structure of
acentric anhydrous beryllium carbonate Be­(CO_3_). Chem. Commun..

[19] Spahr D., Bayarjargal L., Bykova E., Bykov M., Brüning L., Kovalev V., Milman V., Wright J., Winkler B. (2024). 6-Fold-Coordinated
Beryllium in Calcite- Be BeCO_3_] Type. Inorg. Chem..

[20] Bayarjargal L., Spahr D., Milman V., Marquardt J., Giordano N., Winkler B. (2023). Anhydrous aluminium carbonates and
isostructural compounds. Inorg. Chem..

[21] Spahr D., Bayarjargal L., Bykov M., Brüning L., Jurzick P. L., Milman V., Giordano N., Mezouar M., Winkler B. (2024). Synthesis and Characterization
of Lithium Pyrocarbonate
(Li_2_[C_2_O_5_]) and Lithium Hydrogen
Pyrocarbonate (Li­[HC_2_O_5_]). Angew. Chem., Int. Ed..

[22] Aoki K., Yamawaki H., Sakashita M., Gotoh Y., Takemura K. (1994). Crystal Structure
of the High-Pressure Phase of CO_2_ Solid. Science.

[23] Olijnyk H., Jephcoat A. P. (1998). Vibrational studies on CO_2_ up to 40 GPa
by Raman spectroscopy at room temperature. Phys.
Rev. B.

[24] Scelta D., Dziubek K. F., Ende M., Miletich R., Mezouar M., Garbarino G., Bini R. (2021). Extending the Stability Field of
Polymeric Carbon Dioxide Phase V beyond the Earth’s Geotherm. Phys. Rev. Lett..

[25] Yoo C. S., Kohlmann H., Cynn H., Nicol M. F., Iota V., LeBihan T. (2002). Crystal structure of
pseudo-six-fold carbon dioxide
phase II at high pressures and temperatures. Phys. Rev. B.

[26] Datchi F., Giordano F. M., Munsch P., Saitta A. M. (2009). Structure of Carbon
Dioxide Phase IV: Breakdown of the Intermediate Bonding State Scenario. Phys. Rev. Lett..

[27] Brooker M. H., Bates J. B. (1971). Raman and Infrared
Spectral Studies of Anhydrous Li_2_CO_3_ and NaLi_2_CO_3_. J. Chem. Phys..

[28] Milman V., Winkler B. (2001). Prediction of hydrogen
positions in complex structures. Z. Kristallogr..

[29] Pasqualini L. C., Huppertz H., Je M., Choi H., Bruns J. (2021). Triple-Vertex
Linkage of (BO_4_)-Tetrahedra in a Borosulfate: Synthesis,
Crystal Structure, and Quantum-Chemical Investigation of Sr­[B_3_O­(SO_4_)_4_(SO_4_H)]. Angew. Chem., Int. Ed..

[30] Hinteregger E., Wurst K., Perfler L., Kraus F., Huppertz H. (2013). High-Pressure
Synthesis and Characterization of the Actinide Borate Phosphate U_2_[BO_4_]­[PO_4_]. Eur.
J. Inorg. Chem..

[31] Choi C. S., Mighell A. D. (1982). Neutron diffraction study of sodium sesquicarbonate
dihydrate. Acta Crystallogr..

[32] O’Bannon E., Beavers C. M., Williams Q. (2014). Trona at extreme
conditions: A pollutant-sequestering
material at high pressures and low temperatures. Am. Mineral..

[33] Roziere J., Roziere-Bories M. T., Williams J. M. (1976). Unusual hydrogen bonds. A neutron
diffraction study of the hydrogen dinitrate ion, (O_2_NO.H.ONO_2_)^−^, in cesium hydrogen dinitrate. Inorg. Chem..

[34] Huang K.-L., Li G.-J., He Y.-T., Huang R.-D., Pan W.-L., Hu C.-W. (2007). Ln­[(μ_2_-H)­(H-mpdc)_2_]­(H-mpdc)_2_: Lanthanide infinite architectures with symmetric O–H–O
hydrogen bonds (mpdc = 2,6-dimethylpyridine-3,5-dicarboxylate). J. Mol. Struct..

[35] Sano-Furukawa A., Hattori T., Komatsu K., Kagi H., Nagai T., Molaison J. J., dos Santos A. M., Tulk C. A. (2018). Direct observation
of symmetrization of hydrogen bond in *δ*-AlOOH
under mantle conditions using neutron diffraction. Sci. Rep..

[36] Goncharov A. F., Struzhkin V. V., Mao H. -k., Hemley R. J. (1999). Raman Spectroscopy
of Dense *H*
_2_
*O* and the
Transition to Symmetric Hydrogen Bonds. Phys.
Rev. Lett..

[37] Nakamoto K., Margoshes M., Rundle R. E. (1955). Stretching Frequencies as a Function
of Distances in Hydrogen Bonds. J. Am. Chem.
Soc..

[38] Bayarjargal L., Spahr D., Bykova E., Wang Y., Giordano N., Milman V., Winkler B. (2024). High-Pressure Synthesis of an Iron
Carbonate, Fe_2_[CO_3_]_3_. Inorg. Chem..

[39] Wang Y., Bayarjargal L., Bykov M., Bykova E., Spahr D., Glazyrin K., Milman V., Winkler B. (2025). Cr^3+^-Containing
Carbonates and Cr_2_O_3_-*Pbcn* at
Extreme Conditions. Inorg. Chem..

