# Supplementary material for: B[μ-H(CO3)2]: An Acentric Boron Hydrogencarbonate with [μ-H(CO3)2]3– Complex Anions
Source: Inorg Chem. 2025 Sep 16;64(38):19146–50. doi: 10.1021/acs.inorgchem.5c03153 (PMC12486196; doi:10.1021/acs.inorgchem.5c03153)
Supplement: Supplementary file 1 [file ic5c03153_si_001.pdf]

Supplementary material: B[μ-H(CO_3)$_2$]: An acentric boron hydrogencarbonate with [μ-H(CO_3)$_2$]$^{3-}$ complex anions

Dominik Spahr*[,a], Tim H. Reuter[a], Elena Bykova[a], Lkhamsuren Bayarjargal[a], Lukas Brüning[b], Valentin Kovalev[a], Maxim Bykov[b], Lena Wedek[a], Victor Milman[b], Jonathan Wright[d], Björn Winkler[a]

[a]*Goethe University Frankfurt, Institute of Geosciences, Altenhöferallee 1, 60438 Frankfurt, Germany*
[b]*Goethe University Frankfurt, Institute of Inorganic and Analytical Chemistry, Max-von-Laue-Straße 7, 60438 Frankfurt, Germany*
[c]*Dassault Systèmes BIOVIA, 334 Cambridge Science Park, Cambridge CB4 0WN, United Kingdom*
[d]*European Synchrotron Radiation Facility ESRF, 71 avenue des Martyrs, CS40220, 38043 Grenoble Cedex 9, France*

1. Methods

1.1. *Sample material*

The high-pressure experiments were carried out using boron trioxide (B_2O_3) powder (99.98% purity, Merck KGaA, Darmstadt, Germany) as starting material. The B_2O_3 powder was dried at $\approx$ 423 K in a drying oven for 8 h prior the loading of the DAC. The CO_2 gas for the gas-jet was used as purchased (Nippon gases, purity $\geq$ 99.995%). In previous experiments, we found that sometimes small amounts of H_2O from the CO_2 gas co-condense into the gasket hole during the cryogenic loading.[1]

1.2. *High-pressure experiments*

The high-pressure experiments were carried out in Boehler-Almax type DACs equipped with diamonds having an opening angle of 70° and 300 µm sized culets on both sides.[2] We used Re-gaskets, which were preindented to thicknesses of $\approx$ 45 µm. Afterwards, sample chambers with $\approx$ 80 µm diameter were drilled into the Re-gaskets using a custom-built laser set-up. In the next step, a B_2O_3 piece with dimensions of $\approx 50 \times 30 \times 20$ µm^3 was placed on the culet of the bottom diamond. In addition, we placed a ruby chip for pressure determination on the bottom diamond. Dry-ice (CO_2-I) was directly condensed into the sample chamber using a custom-built cryogenic loading system (see Spahr *et al.*[3]). The DAC was opened and placed on a liquid nitrogen cooled Cu-holder, which was cooled down to $\approx$ 100 K. We used a small nozzle to direct the CO_2 gas jet with 5 l min^{-1} directly at the gap between the upper diamond and the gasket. The precipitation of the CO_2 in the gasket hole was monitored using an optical microscope and a camera. After a sufficient amount of CO_2 was deposited in the gasket hole, the DAC was tightly closed. The pressure during compression of the DAC was determined by measuring the shift of the ruby fluorescence and we assume an error of 6% due to non-hydrostatic conditions.[4] We expect that the pressure conditions in the DAC before laser-heating are very likely non-hydrostatic as CO_2-III may sustain pressure gradients up to 0.2 GPa µm^{-1} at high pressures without heating.[5]

1.3. *Laser heating*

The B_2O_3 + CO_2 + H_2O mixture was laser-heated from both sides using a custom-built set-up equipped with a Coherent Diamond K-250 pulsed CO_2 laser (λ = 10600 nm).[6] The laser power was adjusted to achieve a coupling of the laser to the sample, using a laser power between 1–6 W. The maximum temperature achieved during the laser-heating was $T_{\max} = 1500(200)$ K. The temperatures were determined by the two-color pyrometer method, employing Planck and Wien fits.[7] The heating time during the experiments was $\approx$ 30 min. It is well established that laser-heating in DACs always suffers from large temperature gradients and the actual temperature is strongly dependent on the coupling of the laser with the sample, especially at lower temperatures. We estimate an uncertainty of at least ±15% of the nominal temperature in the laser-heated region depending on the focus of the laser beam, based on typical 2D temperature-gradient determination experiments performed in DACs.[8]

1.4. *Raman spectroscopy*

High-pressure Raman spectroscopy was performed in the DACs using an Oxford Instruments WITec alpha 300R Raman imaging microscope. The Raman microscope was equipped with an Olympus SLMPan N 50× objective. The measurements were performed using the 532 nm laser. We employed the 1800 grooves mm^{-1} grating of the WITec UHTS 300S (VIS-NIR) spectrograph in combination with an Andor DR316B-LDC-DD CCD detector for the measurements. The laser power was 100 mW on the sample and the spot size of the Raman laser was $\approx$ 0.8 µm. We assume a depth resolution of $\approx$ 6 µm in the direction of the laser beam. Raman maps were measured on a grid with a step-size of 1 µm. The background of the Raman spectra was corrected using the software package Fityk.[9]

*d.spahr@kristall.uni-frankfurt.de

1.5. *Single-crystal synchrotron X-ray diffraction*

High-pressure single-crystal synchrotron X-ray diffraction was carried out at the ESRF in Grenoble, France, at the Materials Science Beamline line ID11.[10] The beam size on the sample was 0.6×0.6 μm^2. The diffraction data were collected using an Eiger2 X 4M CdTe detector, a wavelength of 0.2846 Å (43.6 keV) and a detector to sample distance of 152 mm. We rotated the DAC by ±34° around the vertical axis perpendicular to the beam while collecting frames in 0.25° steps with 2 s acquisition time per frame.

The detector to sample distance was calibrated using the powder diffraction pattern of a CeO_2 standard in conjunction with the software DIOPTAS.[11] The diffractometer/detector geometry for the analysis of the single-crystal diffraction data was calibrated using diffraction data collected from a silicon single-crystal at ambient pressure. After the data collection, the reflections were indexed and integrated employing CrysAlis^{PRO} (version 43.67a).[12] We used the Domain Auto Finder program (DAFi) to find possible single-crystal domains for the subsequent data reduction.[13] The structure solution and refinement were performed using the software package OLEX2 employing SHELXT for the crystal structure determination and SHELXL for the refinement.[14,15,16]

1.6. *Second harmonic generation measurements*

Second harmonic generation measurements were performed using a custom-built set-up.[17] For the generation of the fundamental pump wave we used an Impex High-Tech Q-switched Nd:YAG laser (1064 nm, 5–6 ns, 2 kHz). The fundamental infrared light was separated from the generated second harmonic (532 nm) with a harmonic separator, a short-pass filter, and an interference filter. The SHG signal was collected using a photomultiplier tube (Hamamatsu R2949) in combination with an oscilloscope (Tektronix TDS2022). The samples were measured in transmission geometry using the Kurtz-Perry approach.[18] We employed quartz (SiO_2) and corundum (Al_2O_3) powders outside a DAC at ambient conditions as reference materials. For measurements in the DAC and of the reference materials 40 individual SHG measurements were carried out and averaged. Background signals between the laser pulses were used to normalize the measured SHG intensities using a Matlab script.

1.7. *Density functional theory-based calculations*

First-principles calculations were carried out within the framework of density functional theory, employing the Perdew-Burke-Ernzerhof (PBE) exchange-correlation functional and the plane wave/pseudopotential approach implemented in the CASTEP simulation package.[19,20,21] "On the fly" norm-conserving or ultrasoft pseudopotentials generated using the descriptors in the CASTEP data base were employed in conjunction with plane waves up to a kinetic energy cutoff of 1020 eV or 630 eV, for norm-conserving and ultrasoft pseudopotentials, respectively. The accuracy of the pseudopotentials is well established.[22] A correction scheme for van der Waals (v.d.W.) interactions was applied in the DFT-calculations. We employed the correction scheme developed by Tkatchenko and Scheffler.[23] A Monkhorst-Pack grid was used for Brillouin zone integrations.[24] We used a distance between grid points of <0.023 Å^{-1}. Convergence criteria for geometry optimization included an energy change of $<5 \times 10^{-6}$ eV atom^{-1} between steps, a maximal force of <0.008 eV Å^{-1} and a maximal component of the stress tensor <0.02 GPa. Phonon frequencies were obtained from density functional perturbation theory (DFPT) calculations.[25,26] Raman intensities were computed using DFPT with the "$2n+1$" theorem approach.[27] The reliability of calculations of SHG tensors has been established previously.[28]

2. Results

2.1. *Single-crystal synchrotron X-ray diffraction*

In order to determine the structure of the unknown phase, we employed synchrotron X-ray diffraction in the area of the gasket hole where we observed mainly Raman modes of the unknown phase. In a first step, we collected X-ray diffraction data on a grid using a spot size of $0.6 \times 0.6\ \mu m^2$ in order to locate promising positions for the collection of single-crystal diffraction data. In the second step, we collected diffraction data suitable for single-crystal X-ray diffraction analysis on selected locations. Fig. S 1 a shows a part of a reciprocal space reconstruction for the ($h1l$) and the ($h0l$) plane on the selected location for the single-crystal structure solution.

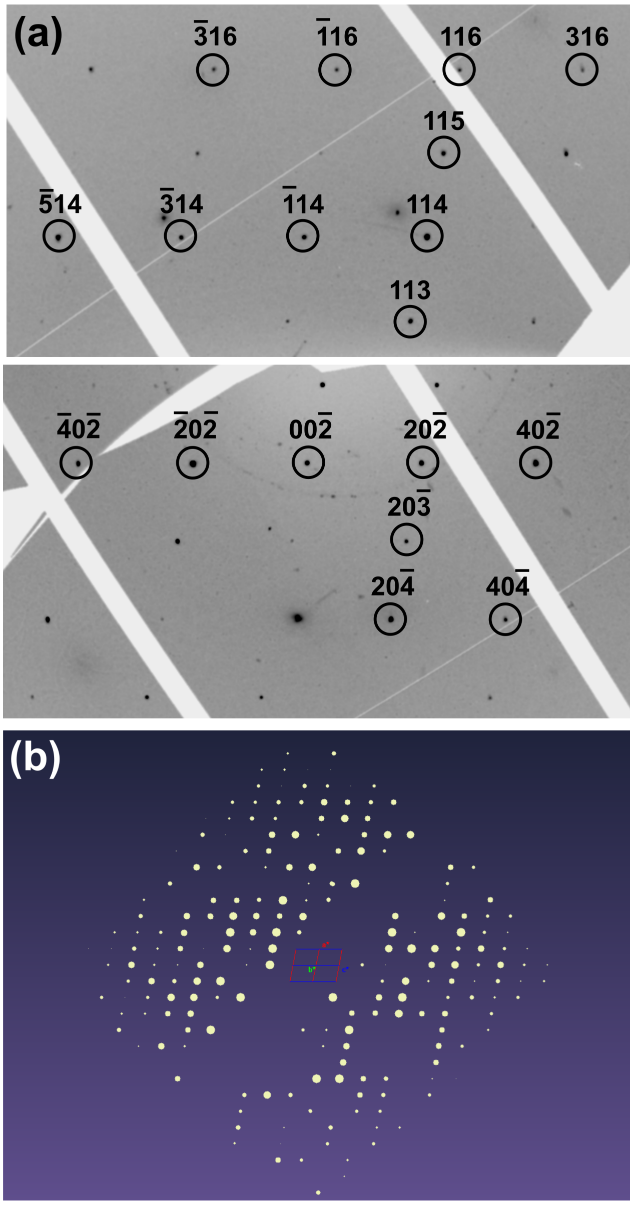

Figure S 1: (a) Reciprocal space reconstruction for the ($h1l$) plane (top) and the ($h0l$) plane (bottom). (b) Schematic depiction of the reflections in reciprocal space in the Ewald-Explorer in CrysAlis. Projection of the reciprocal space is shown along b^* and the intensity of an individual reflection is indicated by the size of the circle.

Besides the reflection of the unknown carbonate phase, we observed reflections and powder rings of different CO_2 phases, of B_2O_3 and of diamond in the diffraction data (Fig. S 1 a). Fig. S 1 b shows the projection of the reciprocal space along b^*. The effect of the shading of diffracted beams due to the metallic body of the DAC and the effect of the inactive detector-areas due to the segments of the Eiger2 X detector can be observed.

Table S 1: Structural parameters of B[μ-H(CO_3)$_2$] at 20(2) GPa from single-crystal structure solution (ambient temperature) in comparison to data from DFT calculations at the same pressure (athermal limit).

	**Single Crystal**	**DFT**
**Crystal data**		
Crystal system	Monoclinic	Triclinic
Space group	$C2$	$P1^*$
Chemical formula	B[μ-H(CO_3)$_2$]	B[μ-H(CO_3)$_2$]
M_r	131.84	131.84
a (Å)	6.997(2)	6.9923
b (Å)	3.868(7)	4.0596
c (Å)	5.0197(8)	5.0824
α (°)	90.0	90.005
β (°)	101.14(3)	99.581
γ (°)	90.0	90.003
V (Å^3)	133.3(2)	142.3
Z	2	2
**Data collection**		-
F_{000}	132	-
θ range (°)	2.62–17.96	-
measured reflections	395	-
independent reflections	272	-
reflections $I > 2\sigma(I)$	246	-
R_{int}	0.016	-
		-
**Refinement**		-
$R_1[I > 2\sigma(I)]$, $wR_2(I)$	0.042, 0.113	-
No. of reflections	272	-
No. of parameters	33	-
No. of restraints	2	-
No. of constraints	1	-
$\Delta\rho_{max}$, $\Delta\rho_{min}$ (e Å^{-3})	0.31, −0.30	-

*symmetry analysis after the DFT-based geometry optimization suggests space group $C2$

The crystal structure of the unknown carbonate phase was solved in acentric space group $C2$ (No. 5) with $Z = 2$ from the collected single-crystal diffraction data. The unknown carbonate has a chemical composition of B[μ-H(CO_3)$_2$]. The R_1-value of 4.2% for the refinement is satisfactory, while the reflection to parameter ratio is reasonable (8.2:1) for a high-pressure DAC experiment. The very low R_{int} (1.6%) and the high $F^2/\sigma(F^2)$-value ($\approx$ 85 up to 0.8 Å and $\approx$ 40 up to 0.5 Å) indicate a very high quality of the experimental data and the data reduction, even if only light elements (H, B, C, O) are present in the crystal structure and the diffraction experiment was performed on a multi-grain reaction product inside a DAC at high pressure.

The displacement parameters of the boron, the carbon and the oxygen atoms were refined anisotropically. In order to reduce the number of free parameters the displacement parameters of the three oxygen atoms were constrained to be identical. The displacement parameter of the hydrogen atom was refined isotropically without constraints. We introduced a distance restraint in order to ensure that the O–H bond length is in agreement with the value derived from the DFT calculations. The position of the hydrogen atom could directly be located in the difference Fourier map, independently whether the displacement parameters of the oxygen atoms were refined isotropically and independent of each other, or anisotropically, but constrained to be equal. The crystallographic parameters of B[μ-H(CO_3)$_2$] derived from the single-crystal structure solution at 20(2) GPa are listed in Table S 1 and in comparison to data derived from the DFT calculations at the same pressure. Atomic coordinates of B[μ-H(CO_3)$_2$] at 20(2) GPa are listed in Table S 2. The anisotropic displacement parameters of the boron, the carbon and the oxygen atoms can be found in the cif-file.

Table S 2: Atomic coordinates and isotropic displacement parameters of B[μ-H(CO_3)$_2$] at 20(2) GPa obtained by single crystal structure refinement (1st line, space group *C*2) and from DFT-based calculations (2nd line, space group *P*1).

**Atom**	**Site**	*x*	*y*	*z*	U_{eq} (Å^2)
B1	2*b*	1	0.416(3)	1/2	0.016(3)
		1.0016	0.4199	0.5017	
C1	4*c*	0.6802(3)	0.299(2)	0.2565(4)	0.009(2)
		0.6822	0.2940	0.2599	
O1	4*c*	0.8414(2)	0.189(1)	0.3971(3)	0.0139(8)
		0.8430	0.2000	0.4032	
O2	4*c*	0.5272(2)	0.128(1)	0.2731(3)	0.0139(8)
		0.5311	0.1200	0.2766	
O3	4*c*	0.6670(2)	0.557(1)	0.1073(3)	0.0139(8)
		0.6668	0.5348	0.1071	
H3	2*a*	1/2	0.62(3)	0	0.04(2)
		0.5017	0.5493	0.0019	

The DFT-based calculations had been carried out in the non-centrosymmetric triclinic space group *P*1 (No. 1) to ensure that no symmetry restrictions were applied to the position of the hydrogen atom. Nevertheless, the theoretical structural model of the borocarbonate B[μ-H(CO_3)$_2$] reproduces the experimental one within the expected errors. A symmetry analysis shows that after geometry optimization the structural model from the DFT calculations has space group symmetry *C*2 within a tolerance of 0.002 Å. The space group symmetry of geometry-optimized structures were analyzed using the software Materials Studio.[29]

2.2. *Pressure dependent DFT-calculations on B[μ-H(CO_3)$_2$]*

We carried out pressure-dependent DFT-calculations on B[μ-H(CO_3)$_2$]. The calculations were carried out in space group *P*1 in order not to constrain the hydrogen position by symmetry. A symmetry analysis after geometry-optimization showed that at pressures > 9 GPa the structure had symmetry *C*2, while for lower pressures and a conventional hydrogen bond the symmetry was *P*1. In addition, our calculations show that at pressures above ≈ 9 GPa the O–H–O geometry is symmetrical with two essentially identical O–H bond distances (Fig. S̃reffig:B-EoS). In contrast, the O–H–O geometry becomes asymmetric at lower pressures, with a clear distinction between the acceptor and the donor oxygen atom (O$\cdots$H–O).

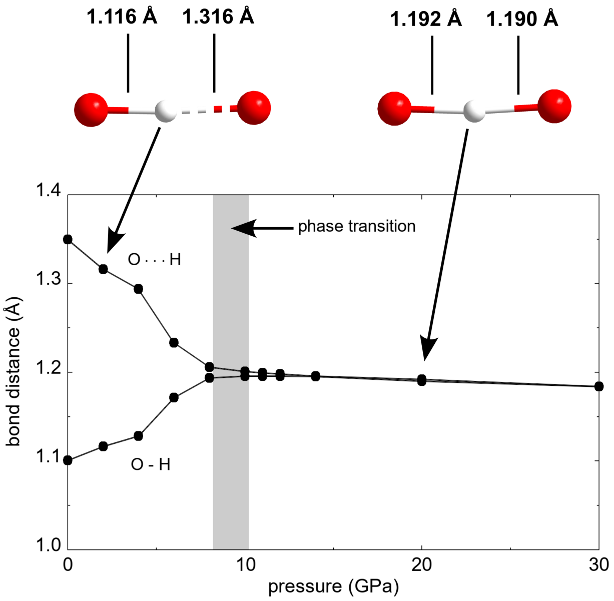

Figure S 2: Pressure dependence of the O–H and O$\cdots$H bond distances in the [μ-H(CO_3)$_2$]$^{3-}$-group of B[μ-H(CO_3)$_2$] from DFT calculations.

We employed DFT-based calculations in the pressure range between 0 GPa and 30 GPa. In order to determine the bulk modulus and its derivative for B[μ-H(CO_3)$_2$] we fitted a 3rd-order Birch-Murnaghan equation of states (EoS) to the calculated *p*, *V*-data using the software package EOSFit7-GUI (Fig. S 3).[30,31,32]

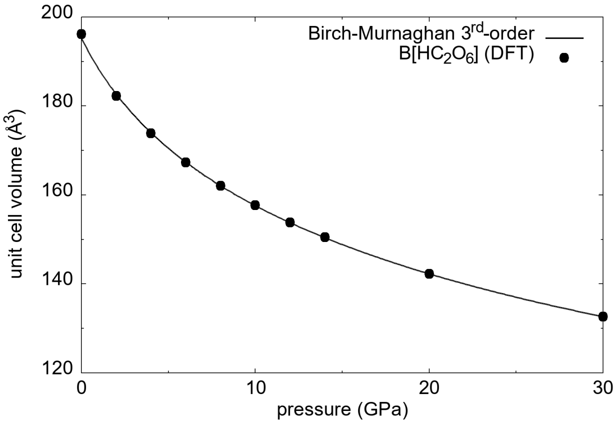

Figure S 3: A Birch-Murnaghan EoS was fitted to the *p*, *V*-data (0–30 GPa) of B[μ-H(CO_3)$_2$] obtained by DFT calculations.

The theoretical bulk modulus of B[μ-H(CO_3)$_2$] and its derivative in the pressure range between 0 GPa and 30 GPa are $K_0 = 24.4(6)$ GPa with $K_p = 6.1(1)$. In addition, we fitted the *p*, *V* data in the range above the phase

transition (10–30 GPa) and found that the compression behavior of B[μ-H(CO_3)$_2$] is not affected by the changes in the O–H–O bond and the accompanied change of the space group symmetry. The EoS-fit results in the same value for the bulk modulus within the expected uncertainties ($K_0 = 25(1)$ GPa with $K_p = 5.9(1)$).

2.3. *Elastic stiffness coefficients*

Elastic stiffens coefficients (c_{ij}) for B[μ-H(CO_3)$_2$] at 0 GPa were computed by the stress strain-method. The tensor components from DFT-based calculations are listed in Table S 3. The bulk modulus computed from the elastic stiffness tensor ranges from 18 GPa (Reuss) to 50 GPa (Hill), depending which averaging scheme is employed. As all eigenvalues of the stiffness matrix are positive, the structure is stable with respect to small distortions, which is a prerequisite for recovery to ambient conditions.

Table S 3: Elastic stiffness coefficients (c_{ij}) for B[μ-H(CO_3)$_2$] at 0 GPa obtained by DFT-based strain-stress calculations.

c_{ij}	DFT calculations (GPa)
c_{11}	173.71(9)
c_{22}	51.44(1)
c_{33}	37.1(6)
c_{44}	20.7(7)
c_{55}	54.2(4)
c_{66}	70.3(4)
c_{12}	51.3(5)
c_{13}	42.0(7)
c_{14}	2.7(3)
c_{15}	37.4(3)
c_{16}	-5.0(2)
c_{23}	3.5(2)
c_{24}	-0.7(2)
c_{25}	-19.6(3)
c_{26}	-0.7(2)
c_{34}	0.7(2)
c_{35}	29.7(4)
c_{36}	-2.2(3)
c_{45}	2.6(2)
c_{46}	-7.7(1)
c_{56}	-3.4(2)

2.4. *Second harmonic generation*

We used our DFT-based calculations to derive the complete SHG tensor for B[μ-H(CO_3)$_2$]. Our calculations reveal a relatively high effective SHG coefficient of $d_{\mathrm{eff}} = 1.8$ pm V^{-1} at 20 GPa.

$$d_{20\,\mathrm{GPa}} = \begin{pmatrix} 0.000 & 0.000 & 0.000 & 0.996 & 0.000 & -2.191 \\ -2.191 & 1.544 & 1.502 & 0.000 & 0.996 & 0.000 \\ 0.000 & 0.000 & 0.000 & 1.502 & 0.000 & 0.996 \end{pmatrix}$$

This coefficient is significantly larger than for quartz ($d_{\mathrm{eff}} = 0.2$ pm V^{-1}), employed as a reference material outside a DAC here ($\approx$ 140 mV). As DFT-GGA-PBE calculations underestimate the band gap, the calculated tensor components will be slightly too large.[28]

It is clear to see that no SHG signal can be observed from the centrosymmetric corundum powder outside a DAC and the unheated B_2O_3 + CO_2 mixture before the laser-heating measured inside the DAC at 20 GPa (Fig. S 4). In addition, we performed second harmonic generation (SHG) measurements at 20(2) GPa in order to confirm the acentric space group symmetry (C2) of B[μ-H(CO_3)$_2$] after laser-heating. We measured a noticeable SHG signal ($\approx$ 25 mV) from the sample in the DAC after the synthesis at 20(2) GPa (Fig. S 4), which is consistent with our DFT calculations showing a relatively high effective SHG coefficient.

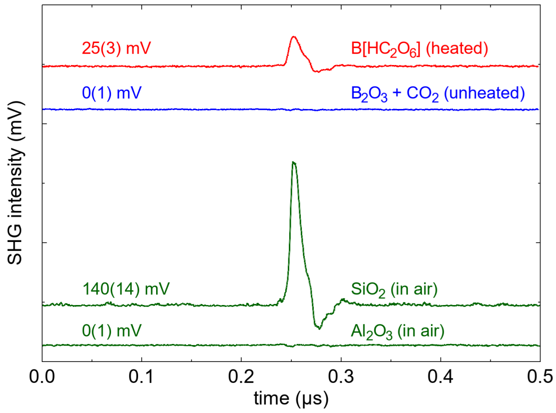

Figure S 4: SHG measurements of B[μ-H(CO_3)$_2$] (red) and the unheated B_2O_3 + CO_2 mixture (blue) at 20(2) GPa in a DAC. Powders of quartz (SiO_2) and corundum (Al_2O_3) where measured outside of the DAC (green) for comparison.

2.5. *Eigenvectors of the atomic displacements*

We used our structural model for DFPT calculations and calculated selected eigenvectors of the atomic displacements in B[μ-H(CO_3)$_2$]. An example of the displacements in the [μ-H(CO_3)$_2$]$^{3-}$ complex anion for the characteristic Raman mode at $\approx$ 1903 cm^{-1} at 0 GPa is shown in Fig S 5.

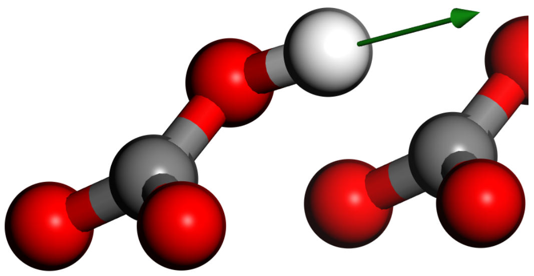

Figure S 5: Eigenvector of the atomic displacements in the [μ-H(CO_3)$_2$]$^{3-}$ complex anion of B[μ-H(CO_3)$_2$] at 0 GPa for the characteristic Raman mode at $\approx$ 1903 cm^{-1}.

References

(1) Spahr, D.; Bayarjargal, L.; Bykov, M.; Brünning, L.; Jurzick, P. L.; Milman, V.; Giordano, N.; Mezouar, M.; Winkler, B. Synthesis and Characterization of Lithium Pyrocarbonate ($Li_2[C_2O_5]$) and Lithium Hydrogen Pyrocarbonate ($Li[HC_2O_5]$). *Angew. Chem. Int. Ed.* **2024**, *63*, e202409822, DOI: 10.1002/anie.202409822

(2) Boehler, R. New diamond cell for single–crystal X-ray diffraction. *Rev. Sci. Instrum.* **2006**, *77*, 115103–1–115103–3, DOI: 10.1029/JB091iB05p04673

(3) Spahr, D.; König, J.; Bayarjargal, L.; Luchitskaia, R.; Milman, V.; Perlov, A.; Liermann, H.-P.; Winkler, B.; Synthesis and Structure of $Pb[C_2O_5]$: An Inorganic Pyrocarbonate Salt. *Inorg. Chem.* **2022**, *61*, 9855–9859, DOI: 10.1021/acs.inorgchem.2c01507

(4) Mao, H. K.; Xu, J.; Bell, P. M. Calibration of the ruby pressure gauge to 800 kbar under quasi-hydrostatic conditions. *J. Geophys. Res.* **1986**, *91*, 4673–4676, DOI: 10.1029/JB091iB05p04673

(5) Yoo, C. S.; Cynn, H.; Gygi, F.; Galli, G.; Iota, V.; Nicol, M.; Carlson, S.; Häusermann, D.; Mailhiot, C. Crystal Structure of Carbon Dioxide at High Pressure: "Superhard" Polymeric Carbon Dioxide. *Phys. Rev. Lett.* **1999**, *83*, 5527–5530, DOI: 10.1103/PhysRevLett.83.5527

(6) Bayarjargal, L.; Fruhner, C.-J.; Schrodt, N.; Winkler, B. $CaCO_3$ phase diagram studied with Raman spectroscopy at pressures up to 50 GPa and high temperatures and DFT modeling. *Phys. Earth Planet. Inter.* **2018**, *281*, 31–45, DOI: 10.1016/j.pepi.2018.05.002

(7) Benedetti, L. R.; Loubeyre, P. Temperature gradients, wavelength-dependent emissivity, and accuracy of high and very-high temperatures measured in the laser-heated diamond cell. *High Press. Res.* **2004**, *24*, 423–455, DOI: 10.1080/08957950412331331718

(8) Du, Z.; Amulele, G.; Benedetti, L. R.; Lee, K. K. M. Mapping temperatures and temperature gradients during flash heating in a diamond-anvil cell. *Rev. Sci. Instrum.* **2013**, *84*, 075111, DOI: 10.1063/1.4813704

(9) Wojdyr, M. *Fityk*: a general-purpose peak fitting program. *J. Appl. Cryst.* **2010**, *43*, 1126–1128, DOI: 10.1107/S0021889810030499

(10) Wright, J.; and Giacobbe, C.; and Majku, M. New opportunities at the Materials Science Beamline at ESRF to exploit high energy nano-focus X-ray beams. *Curr. Opin. Solid. St. M.* **2024**, *24*, 100818, DOI: 10.1016/j.cossms.2020.100818

(11) Prescher, C.; Prakapenka, V. B. *DIOPTAS*: a program for reduction of two-dimensional X-ray diffraction data and data exploration. *High. Press. Res.* **2015**, *35*, 223–230, DOI: 10.1080/08957959.2015.1059835

(12) Agilent, CrysAlis PRO, Yarnton, England, **2014**

(13) Aslandukov, A.; Aslandukov, M.; Dubrovinskaia, N.; Dubrovinsky, L. *Domain Auto Finder* (*DAFi*) program: the analysis of single-crystal X-ray diffraction data from polycrystalline sample. *J. Appl. Cryst.* **2022**, *55*, 1383–1391, DOI: 10.1107/S1600576722008081

(14) Dolomanov, O. V.; Bourhis, L. J.; Gildea, R. J.; Howard, J. A. K.; Puschmann, H. *OLEX2*: a complete structure solution, refinement and analysis program. *J. Appl. Cryst.* **2009**, *42*, 339–341, DOI: 10.1107/S0021889808042726

(15) Sheldrick, G. M. *SHELXT* — Integrated space-group and crystal-structure determination. *Acta. Cryst.* **2015**, *A71*, 3–8, DOI: 10.1107/S2053273314026370

(16) Sheldrick, G. M. Crystal structure refinement with *SHELXL*. *Acta. Cryst.* **2015**, *C71*, 3–8, DOI: 10.1107/S2053229614024218

(17) Bayarjargal, L.; Winkler, B. Second harmonic generation measurements at high pressures on powder samples. *Z. Kristallogr.* **2014**, *229*, 92–100, DOI: 10.1515/zkri-2013-1641

(18) Kurtz, S. K.; Perry, T. T. A Powder Technique for the Evaluation of Nonlinear Optical Materials. *J. Appl. Phys.* **1968**, *39*, 3798–3813, DOI: 10.1063/1.1656857

(19) Hohenberg, P.; Kohn, W. Inhomogeneous Electron Gas. *Phys. Rev.* **1967**, *136*, B864–B871, DOI: 10.1103/PhysRev.136.B864

(20) Perdew, J. P.; Burke, K.; Ernzerhof, M. Generalized Gradient Approximation Made Simple. *Phys. Rev. Lett.* **1996**, *77*, 3865–3868, DOI: 10.1103/PhysRevLett.77.3865

(21) Clark, S. J.; Segall, M. D.; Pickard, C. J.; Hasnip, P. J.; Probert, M. I. J.; Refson, K.; Payne, M. C. First principles methods using CASTEP. *Z. Kristallogr.* **2005**, *220*, 567–570, DOI: 10.1524/zkri.220.5.567.65075

(22) Lejaeghere, K.; Bihlmayer, G.; Björkman, T.; Blaha, P.; Blügel, S.; Blum, V.; Caliste, D.; Castelli, I. E.; Clark, S. J.; Dal Corso, A. et al. Reproducibility in density functional theory calculations of solids. *Science* **2016**, *351*, aad3000, DOI: 10.1126/science.aad3000

(23) Tkatchenko, A.; Scheffler, M. Accurate Molecular Van Der Waals Interactions from Ground-State Electron Density and Free-Atom Reference Data. *Phys. Rev. Lett.* **2009**, *102*, 073005, DOI: 10.1103/PhysRevLett.102.073005

(24) Monkhorst, H. J.; Pack, J. D. Special points for Brillouin-zone integrations. *Phys. Rev. B* **1976**, *13*, 5188–5192, DOI: 10.1103/PhysRevB.13.5188

(25) Baroni, S.; de Gironcoli, S.; Dal Corso, A.; Giannozzi, P. Phonons and related crystal properties from density-functional perturbation theory. *Rev. Mod. Phys.* **2001**, *73*, 515–562, DOI: 10.1103/RevModPhys.73.515

(26) Refson, K.; Tulip, P. R.; Clark, S. J. Variational density-functional perturbation theory for dielectrics and

lattice dynamics. *Phys. Rev. B* **2006**, *73*, 155114, DOI: 10.1103/PhysRevB.73.155114

(27) Miwa, K. Prediction of Raman spectra with ultrasoft pseudopotentials. *Phys. Rev. B* **2011**, *84*, 094304, DOI: 10.1103/PhysRevB.84.094304

(28) Bonnin, M. A.; Bayarjargal, L.; Wolf, S.; Milman, V.; Winkler, B.; Feldmann, C. $GaSeCl_5O$: A Molecular Compound with Very Strong SHG Effect. *Inorg. Chem.* **2021**, *60*, 15653–15658, DOI: 10.1021/acs.inorgchem.1c02315

(29) Meunier, M.; Robertson, S. *Materials Studio* 20th anniversary. *Mol. Simul.* **2021**, *47*, 537–539, DOI: 10.1080/08927022.2021.1892093

(30) Murnaghan, F. The Compressibility of Media under Extreme Pressures. *Proc. Natl. Acad. Sci.* **1944**, *30*, 244–247, DOI: 10.1073/pnas.30.9.244

(31) Birch, F. Finite Elastic Strain of Cubic Crystals. *Phys. Rev.* **1947**, *71*, 809–824, DOI: 10.1103/PhysRev.71.809

(32) Gonzalez-Platas, J.; Alvaro, M.; Nestola, F.; Angel, R. *EosFit7-GUI*: a new graphical user interface for equation of state calculations, analyses and teaching. *J. Appl. Cryst.* **2016**, *49*, 1377–1382, DOI: 10.1107/S1600576716008050
